# What are the success rates of anterior restorations used in localised wear cases?

**DOI:** 10.1038/s41432-025-01112-z

**Published:** 2025-02-01

**Authors:** Bryan Murchie, Nikita Jiwan, David Edwards

**Affiliations:** 1 Clinical Lecturer in Restorative Dentistry, School of Dental Sciences, Newcastle University, Newcastle upon Tyne, England; 2 General Dentist at Dental Beauty Basildon & L&Y Dental, Northwood, England; 3 Doctoral Research fellow, School of Dental Sciences, Newcastle University, Newcastle upon Tyne, England

**Keywords:** Dentine, Bonded restorations, Composite resin, Tooth erosion

## Abstract

**Objective:**

A systematic review and meta-analysis of the literature was carried out assessing the success and survival rate of anterior restorations used in localised wear cases. This also included assessment of posterior teeth re-establishing occlusal contact following use of the Dahl approach.

**Data sources:**

Two large databases; Medline via OVID, and Scopus were used to identify existing literature. The review followed Preferred Reporting Items for Systematic Reviews and Meta-Analysis (PRISMA) and Meta-analyses guidelines and used the PIO framework [1]. Grey literature was also searched.

**Study selection:**

Publications written in English were included between Jan 1970 and Nov 2020. Population: participants with localised anterior tooth loss. Intervention: anterior composite restorations. Outcome: success and survival rates of the composite restorations. Six cohort studies were included in the final analysis, with three prospective and three retrospective. These studies evaluated the success and survival rates of direct and indirect composite restorations, with a follow-up period ranging from 5 months to 10 years, which took place between 2000 to 2016.

**Data extraction and synthesis:**

Extracted data included: author(s) and year, study type, number and age of participants, number of restorations, location for intervention, type of intervention (direct vs indirect), type of composite, increase in OVD (amount of increase and period to re-establish posterior occlusion), follow up period, definition of failure, number of failed restorations, assessment of intervention and longevity/survival rate.

Risk of bias in individual studies was assessed using The Newcastle Ottawa quality assessment scale (NOS) for cohort studies. Outcome measures were standardised as success or survival:

- Success – restoration assigned category A on the modified US Public Health Service (USPHS) criteria.
- Survival – restoration assigned category A or B on the modified USPHS criteria.

The restoration was considered a failure if any other grades were allocated on the modified USPHS criteria. Results were visualised using forest plots. Heterogeneity between studies was measured by I-squared statistic. Sensitivity analysis was performed for each outcome.

**Results:**

The survival rates for composite restorations (direct and indirect) at 2–10 years follow-up was 88% (95% CI, 70–90%; I^2^ = 97%). When significant outliers from one study were removed, and re-analysed, the survival rates increased to 93% at 2–7 years follow-up (95% CI, 85–98%; I^2^ = 83%). Success rates for composite restorations over the same 2–10 year period was 68% (95% CI, 44–87%; I^2^ = 98%). Heterogeneity was generally considered high due to large variations in study design, sample size, type of intervention, and follow-up period.

The success of the anterior composite Dahl approach in re-establishing posterior occlusion was reported at 85% (95% CI, 73%–94%), which took between 1.5 and 25.4 months.

**Conclusions:**

Anterior composite restorations had a high success rate over a period of 2–10 years in patients affected by localised tooth wear, which was higher over a 2–7 year interval. Although, the overall survival rates were considerably lower when accounting for minor and major types of restoration failure. This review supported continued use of anterior composites for restoring worn teeth, which had good short-medium term longevity. However, these conclusions should be interpreted with caution considering the quality of evidence, the heterogeneity of the studies and the limited number of studies included.

## A Commentary on

**Aziz I M, Locke M**.

Success and Survival of Composite Resin Restorations for the Management of Localized Anterior Tooth Wear: A Systematic Review and Meta-Analysis. *Eur J Prosthodont Restor Dent* 2024; **32:** 403–414.

**GRADE Rating**: 

## Commentary

The prevalence rates for tooth surface loss have been continually increasing worldwide, impacting all age groups^1^. Recent literature has shown that a large proportion of this can be attributed to erosive tooth wear, where the leading aetiological factor is extrinsic acids contained within dietary foods and beverages^2^. This has led to an increased incidence of pathological tooth wear, where restorative intervention is required. Clinically, this presents as a complete breakdown of the enamel surface, with dentine exposure, leading to a reduced quality of life^3^. Early diagnosis and prevention are key, however, many patients do not present until severe tooth surface loss has already occurred. The current concept of minimally invasive dentistry aims to preserve tooth structure/integrity and pulp vitality, therefore, restoration of the worn dentition should prioritise additive approaches, such as composite build-ups, in favour over more destructive indirect methods for restoring oral health. Although, a better understanding is required of the longevity of direct restorations, especially as indirect restorations remain commonplace as part of clinical management in tooth wear cases.

The primary aim of the present systematic review^4^ was to assess the success and survival rates of anterior composite restorations used in localised tooth wear cases. The secondary aim was to investigate whether the posterior teeth re-established occlusal contact following use of the anterior Dahl approach. The review was initially registered on the International Prospective Register of Systematic Reviews (PROSPERO), ensuring transparency and reducing the risk of bias.

This review used PIO, instead of PICO, to formulate the research question. It could be assumed this was because there were no straight forward control groups. In terms of assessing the restoration success, the modified USPHS criteria were utilised, where the restorations are graded: Alpha, clinically ideal restoration; Bravo, minor deviations from ideal; Charlie, the restoration requires replacement; and Delta, the restoration needs replaced immediately. Although, the use of modified USPHS has decreased in recent years in favour of a newer FDI system, which is thought to provide a higher level of standardisation^5^.

It is possible that some relevant studies were not included in the present study. Firstly, there was a delay of four years from running the database searches to publication, and it may have been desirable to repeat and update the searches prior to submission for publication. Secondly, only two databases were searched (Medline via OVID and Scopus), further limiting the number of studies located for inclusion. The search terms used appeared appropriate and there was a clear outline of the inclusion and exclusion criteria.

Another potential limitation of the present study was that a single author undertook the full screening process, including title and abstract screening followed by full text screening. Conventional best practice involves two authors screening independently with a third author able to resolve any conflicts. This may introduce a potential source of bias and limits the validity of the results.

Seven prospective and retrospective cohort studies were included at the final extraction phase. This was later changed to six studies, as one prospective study was actually a review and not primary research. This highlighted the need for a robust screening process. The included cohort studies are also considered lower quality evidence compared with randomised control trials. However, the internal validity of the studies was good, where all six studies were rated high quality with a low risk of bias, verified by both authors.

The restoration follow-up periods ranged from 5 months to 10 years. The leading aetiology of the tooth surface loss was attributed to erosion in the majority of patient cases, followed by a multifactorial diagnosis (erosion, attrition and abrasion). The level of OVD increase was reported between 0.5 mm and 5 mm. Re-establishment of the posterior teeth after anterior build-ups occurred in 85% of cases, which took between 1.5 to 25.4 months. A direct comparison was attempted between the success of indirect and direct composites, however, calculations showed too much heterogeneity which limited the reliability of the results. Interestingly, the use of indirect anterior composite restorations was only included by the comparatively older studies, dated between 14 and 23 years old. Only one cohort study in this review was published within the last 10 years, the remaining studies could be considered outdated in terms of dental materials currently available.

The overall success rates of composite restorations on anterior teeth were 88-93%, over a period of 2–10 years, with consideration of major failures only (restorations that were lost, broken, or clinically unacceptable). When this same time period also encompassed failures (restorations requiring replacement or urgent replacement), the success rates dropped to 68%. Although, these findings should be interpretated with general caution due to the significant heterogeneity between different studies.

## Summary

The present systematic review demonstrated relatively high success rates when anterior composite restorations were used to manage localised wear cases, where posterior teeth re-established occlusal contact in 85% of cases. However, the overall findings and conclusions from this review should be interpreted with caution. The methods did not follow conventional best practice in terms of study selection, which introduced uncertainty of the included publications. The delay from running searches to publishing also means that more recent studies were not included. The majority of cohort studies were also published >10 years prior, meaning there is lower relevance to more recent composite systems. Additional primary research is required to help better understand the longevity of composite restorations for use in wear cases, analysed using a well-designed systematic review.

## Practice points

- Localised anterior wear cases can be successfully managed by composite restorations with an overall high success rate in the short-medium term.
- In 85% of cases, posterior teeth will re-establish occlusal contact within two years following placement of anterior restorations that increased the OVD up to 5 mm.
